# Molecular prevalence and subtyping of *Cryptosporidium hominis* among captive long-tailed macaques (*Macaca fascicularis*) and rhesus macaques (*Macaca mulatta*) from Hainan Island, southern China

**DOI:** 10.1186/s13071-019-3449-0

**Published:** 2019-04-30

**Authors:** Wei Zhao, Huanhuan Zhou, Hairong Jin, Meicen Liu, Mingyan Qiu, Lihua Li, Feifei Yin, Jasper Fuk-Woo Chan, Gang Lu

**Affiliations:** 1 Department of Pathogenic Biology, Hainan Medical University, Haikou, Hainan China; 2 Hainan Medical University-The University of Hong Kong Joint Laboratory of Tropical Infectious Diseases, Hainan Medical University, Haikou, Hainan China; 3 Key Laboratory of Translation Medicine Tropical Diseases, Hainan Medical University, Haikou, Hainan China; 4 Hainan Jingang Biological Technology Co., Ltd., Haikou, Hainan China; 5 Department of Microbiology, Li Ka Shing Faculty of Medicine, The University of Hong Kong, Pokfulam, Hong Kong Special Administrative Region China

**Keywords:** *Cryptosporidium*, *Macaca fascicularis*, *Macaca mulatta*, Gp60, *SSU* rRNA

## Abstract

**Background:**

*Cryptosporidium* is an important zoonotic parasite that is commonly found in non-human primates (NHPs). Consequently, there is the potential for transmission of this pathogen from NHPs to humans. However, molecular characterization of the isolates of *Cryptosporidium* from NHPs remains relatively poor. The aim of the present work was to (i) determine the prevalence; and (ii) perform a genetic characterization of the *Cryptosporidium* isolated from captive *Macaca fascicularis* and *M. mulatta* on Hainan Island in southern China.

**Methods:**

A total of 223 fresh fecal samples were collected from captive *M. fascicularis* (*n* = 193) and *M. mulatta* (*n* = 30). The fecal specimens were examined for the presence of *Cryptosporidium* spp. by polymerase chain reaction (PCR) and sequencing of the partial small subunit (*SSU*) rRNA gene. The *Cryptosporidium*-positive specimens were subtyped by analyzing the 60-kDa glycoprotein (*gp60*) gene sequence.

**Results:**

*Cryptosporidium* spp. were detected in 5.7% (11/193) of *M. fascicularis*. All of the 11 *Cryptosporidium* isolates were identified as *C. hominis*. Subtyping of nine of these isolates identified four unique *gp60* subtypes of *C. hominis.* These included IaA20R3a (*n* = 1), IoA17a (*n* = 1), IoA17b (*n* = 1), and IiA17 (*n* = 6). Notably, subtypes IaA20R3a, IoA17a, and IoA17b were novel subtypes which have not been reported previously.

**Conclusions:**

To our knowledge, this is the first reported detection of *Cryptosporidium* in captive *M. fascicularis* from Hainan Island. The molecular characteristics and subtypes of the isolates here provide novel insights into the genotypic variation in *C. hominis*.

## Background

*Cryptosporidium* is a protozoan belonging to the phylum Apicomplexa. The parasite is the causative agent of cryptosporidiosis, the clinical signs of which include diarrhea, malabsorption and wasting in humans [1]. Cryptosporidiosis is a significant threat to immunocompromised patients, especially among patients with human immunodeficiency virus (HIV)/ Acquired Immunodeficiency Syndrome (AIDS) in whom the mortality rate is high [2]. Cryptosporidiosis in children is associated with malnutrition and poor growth and is one of the most important causes of diarrhea-associated death among young children in developing countries [3]. In addition to humans, epidemiological evidence showed that *Cryptosporidium* is capable of infecting more than 260 vertebrate species, including mammals, birds, reptiles, fish and amphibians [4, 5]. *Cryptosporidium* oocysts are ubiquitous in the environment and more than 550 water-borne and food-borne outbreaks of cryptosporidiosis have been reported globally, with the sources of infection linked to drinking or recreational water, fruits, vegetables, or cow’s milk [6, 7]. Because of the clinical and public health importance of *Cryptosporidium*, this protozoan is considered as a category B list priority pathogen by the National Institutes of Health (NIH) of the USA and the fifth most important food-borne parasite by the Food and Agriculture Organization (FAO)/World Health Organization (WHO) Expert Committee [8, 9].

Extensive genetic variations have been reported within the genus *Cryptosporidium*. Thus far, 38 species and more than 40 genotypes have been identified [10]. Over 20 species or genotypes of *Cryptosporidium* have been found in humans [10]. Most human-pathogenic *Cryptosporidium* species and genotypes have also been found in animals [10]. The accurate identification of *Cryptosporidium* in animals at the species and/or genotype level is essential for the assessment of the potential zoonotic sources of infection among humans [10]. The two most common species detected in humans, *C. parvum* and *C. hominis*, are responsible for > 90% of human cases of cryptosporidiosis worldwide, and are responsible for almost all outbreaks of cryptosporidiosis [11]. While *C. parvum* is generally accepted to be a zoonotic pathogen and it was widely accepted that cases of *C. hominis* were transmitted from human-to-human, but recent reports have shown that *C. hominis* are more commonly found in animals (including NHPs, horses, and donkeys) [10]. Molecular subtyping has been increasingly used to study the transmission of *Cryptosporidium* in human and animals. Several subtypes of *C. hominis* and *C. parvum* have been identified based on the 60 kDa glycoprotein (*gp60*) gene sequence, which is the most commonly used genetic locus for the subtyping of *Cryptosporidium* [12]. These subtyping results have shown that the same subtypes of *C. parvum* may be found in humans and their epidemiologically-linked animals, suggesting that infected animals are a major source of human infection [13, 14].

Among animals, NHPs, due to their high level of genetic homology to humans, are invaluable experimental models for biomedical research. In addition, they may be susceptible to infection with numerous human pathogens including *Cryptosporidium* [15]. More than 40 studies from 12 countries have been published describing infection of NHPs with *Cryptosporidium*. However, few studies have included data on genotyping [16]. Eight species of *Cryptosporidium* have been reported in non-human primates including *C. hominis*, *C. parvum*, *C. felis*, *C. muris*, *C. ubiquitum*, *C. meleagridis*, *C. bovis* and *C. andersoni* [15, 17–29]. Interestingly, all of these species have been detected in humans as well. *Macaca fascicularis* (long-tailed macaque) and *M. mulatta* (rhesus macaque) are two common species of NHPs which live in close proximity to many humans, and frequently interact with human communities in many locations, including China. The health of *M. fascicularis* and *M. mulatta* are therefore an important public health issue. The aim of the present study was to determine the prevalence of natural *Cryptosporidium* infection in captive *M. fascicularis* and *M. mulatta.* The sampled animals were from a facility that breeds NHPs for research purposes in Hainan Island, China. The second aim of this study was to subtype the *C. hominis* isolates sequencing the *gp60* gene.

## Methods

### Collection of fecal specimens

A total of 223 fresh fecal samples were collected from 193 *M. fascicularis* and 30 *M. mulatta* between July and August 2018 at the breeding base of experimental primates of Hainan Jingang Biological Technology Co., Ltd., at Haikou, Hainan, China. This breeding base of experimental primate was established in 2003. At the time of sample collection, the facility housed over 10,000 animals. All *M. fascicularis* in the facility were reared in groups, with the exception of infants, who were housed alone with their mothers until weaning (at approximately 8 months of age). Young animals aged 1–2 years were kept in individual cages for a quarantine period of 30 days before being sold to research laboratories. Two groups of *M. fascicularis* were sampled in this study: one group contained 125 weaned (one year-old) *M. fascicularis* who were housed individually, and the other group contained 68 adult *M. fascicularis* (> five years of age) who were housed in groups of 20–30 animals per cage for breeding purposes. For singly housed animals, fresh feces were collected from the floor of the cages immediately after defecation. For animals housed in groups, fresh fecal deposits were collected from the ground in the early morning, as the floors of animal houses were cleaned every evening. To minimize the chance of duplicate sampling of animals, only one fecal specimen was collected at one location of the ground in each animal pen within any house each time. The *M. mulatta* sampled in this study were all > 10 years-old and were maintained in individual cages for research laboratories. All the fecal samples were put into individual plastic bags marked with the age and health status of each animal. All samples were transported to our laboratory in a cooler with ice packs within 24 h and were stored at 4 °C until the time of processing.

### DNA extraction

Prior to DNA extraction, all fecal specimens were processed by filtering through mesh to remove large solids, concentrating, and washing three times with distilled water by centrifugation for 10 min at 1500×*g*. Genomic DNA was extracted using a QIAamp DNA Stool Mini Kit (Qiagen, Hilden, Germany) according to the manufacturer’s instructions. DNA was eluted in 200 ml of Buffer AE and stored at − 20 °C prior to use in PCR analysis.

### *Cryptosporidium* genotyping and subtyping

All DNA preparations were tested for the presence of *Cryptosporidium* spp. by nested PCR amplification of an 830 bp nucleotide fragment of the *SSU* rRNA gene, using the primers previously described by Xiao et al. [30]. The cycling parameters for the PCR reactions were optimized and used as follows: 94 °C for 3 min and 35 cycles of 95 °C for 30 s, 55 °C for 30 s, and 72 °C for 90 s, followed by a final extension step at 72 °C for 7 min. TaKaRa TaqDNA Polymerase (TaKaRa Bio Inc., Tokyo, Japan) was used for all PCR reactions. A negative control with no DNA was included in all PCR tests. Subtyping of *Cryptosporidium*-positive samples was conducted by nested PCR amplification of fragments of approximately 800–850 bp of the *gp60* gene [31].

### Sequence analysis

All PCR products were sequenced using PCR primers for each locus on an ABI PRISMTM 3730 DNA Analyzer (Applied Biosystems, Carlsbad, CA, USA), in conjunction with the BigDye Terminator v3.1 Cycle Sequencing kit (Applied Biosystems, USA). The accuracy of the sequencing data was confirmed by sequencing in both directions. Nucleotide sequences obtained in the present study were subjected to BLAST searches (http://www.ncbi.nlm.nih.gov/blast/) and then analyzed and aligned with each other and the published reference sequences of *Cryptosporidium* in GenBank, using ClustalX 1.81 (http://www.clustal.org/).

### Statistical analysis

Differences in infection rates among different groups of animals were compared using the Chi-square test or Fisher’s exact test in SPSS 17.0. *P* < 0.05 was considered to be statistically significant.

## Results

### Prevalence rate of *Cryptosporidium* spp. in *M. fascicularis* and *M. mulatta*

Of the 223 fecal samples tested, 11 (4.9%) were found to be positive for *Cryptosporidium* by PCR (Table 1). All 11 *Cryptosporidium*-positive samples were from *M. fascicularis* (11/193, 5.7%) and none was found in *M. mulatta* (0/30, 0%), although this did not reach statistical significance (*P* = 0.37). It was also observed that only young *M. fascicularis* were infected with *Cryptosporidium*. Furthermore, infection was observed in both males and females, as well as in both diarrhea and non-diarrhea groups. Whereas differences in the *Cryptosporidium* infection rates between female (6.5%; 6/92) and male (5.0%; 5/101) *M. fascicularis* were not significant (*P* > 0.05), positive animals with diarrhea (13.8%; 4/29) were significantly higher than without diarrhea (4.3%; 7/164) (*P* = 0.04).

**Table 1:** Prevalence and distribution of *Cryptosporidium* species and subtypes in long-tailed macaque by age, sex and symptom

Group		No. examined	No. positive (%)	*Cryptosporidium* species (*n*)	Subtype (*n*)
Age	Young	125	11 (8.8)	*C. hominis* (11)	IaA20R3a (1); IoA17a (1); IoA17b (1); IiA17 (6)
	Adult	68	0	–	–
Sex	Female	92	6 (6.5)	*C. hominis* (6)	IiA17 (4)
	Male	101	5 (5.0)	*C. hominis* (5)	IaA20R3a (1); IoA17a (1); IoA17b (1); IiA17 (2)
Symptom	Diarrhea	29	4 (13.8)	*C. hominis* (4)	IaA20R3a (1); IoA17a (1); IoA17b (1); IiA17 (1)
	Non-diarrhea	164	7 (4.3)	*C. hominis* (7)	IiA17 (5)

### Genetic characterization of the *Cryptosporidium* spp. at the *SSU* rRNA and *gp60* loci

**Fig. 1:**
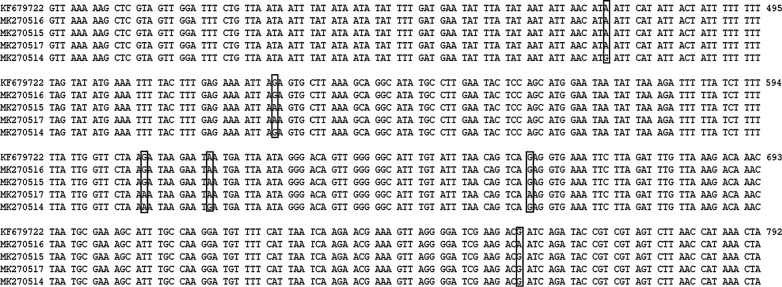
Sequence variation in the small subunit (SSU) rRNA gene among of *C. hominis* isolates. SSU rRNA sequences of one known type (GenBank: KF679722) and four novel types (GenBank: MK270514-MK270517) identified in this study were aligned. The first nucleotide of the reference sequence KF679722 was considered to be position number one

Sequence analysis of 11 PCR products of the *SSU* rRNA gene of *Cryptosporidium* indicated that all 11 samples were *C. hominis*. Furthermore, the obtained sequences represented five types with six polymorphic sites (Fig. 1). One *SSU* rDNA sequence representing seven *Cryptosporidium* isolates was identical to that of the *C. hominis* isolate derived from a rhesus macaque in Henan Province of China (GenBank: KF679722). The other four sequences (GenBank: MK270514-MK270517) had not been reported previously. These sequences had either one (position 528 or 760) or three (positions 475, 609 and 618 or 528, 609 and 661) base variations compared with the sequence KF679722, which had the greatest similarity to the novel sequences obtained here. The first nucleotide of the reference sequence (KF679722) was considered to be position number one.

*Cryptosporidium hominis*-positive specimens were subtyped by sequence analysis of the *gp60* gene. Of the 11 *C. hominis* isolates obtained, nine were successfully amplified. Sequence analysis of the nine *gp60* gene sequences suggested that they belong to three subtype families (Ia, Ii and Io). Four subtypes, IaA20R3a (*n* = 1), IoA17a (*n* = 1), IoA17b (*n* = 1) and IiA17 (*n* = 6), were detected in the present study based on the established nomenclature system of the *gp60* subtypes. Among them, the novel subtypes IaA20R3a, IoA17a and IoA17b have not been reported previously. The sequence of subtype IaA20R3a (MK270518) has a maximum homology of 97% to isolate JF927196 which was isolated from Bangladeshi children. There were three nucleotide differences observed between IoA17a (MK270519) and IoA17b (MK270520). In addition, these isolates shared 98% identity with the sequence (KX926459) of subtype IdA15 which was initially misdiagnosed, and has subsequently been renamed IoA15 [10]. The novel *gp60* subtypes IaA20R3a, IoA17a and IoA17b showed the same rRNA type KF679722. Isolate IiA17 was identical to the sequence of a *C. hominis gp60* subtype isolated from a rhesus macaque in Henan, China (HQ397716). The four novel rRNA types (MK270514-MK270517) showed the same *gp60* subtype IiA17. Subtype IiA17 was isolated from feces in monkeys with diarrhea as well as the non-diarrhea group. In contrast, subtypes IaA20R3a, IoA17a and IoA17b were only detected in the diarrhea group.

## Discussion

In the present study, *Cryptosporidium hominis* was only identified in *M. fascicularis*, with an overall prevalence of 5.7% (11/193). This observation is consistent with the report in captive *M. mulatta* from the Qinling Mountains (7.0%) and lower than that reported in wild *M. mulatta* (10.9%) from Guizhou, China [19, 21]. While the prevalence of *Cryptosporidium* in *M. fascicularis* observed here is higher than in other Chinese primates, another study of 26 NHP species by Karim et al. [20] observed *Cryptosporidium* was detected in 19 of the 2660 (0.7%) faecal specimens tested from China. Interestingly, only four NHP species were infected with *Cryptosporidium* (0.7% of rhesus macaques, 1.0% of cynomolgus monkeys, 10.0% of slow lorises and 6.7% of Francois’ leaf monkeys). Low prevalence of *Cryptosporidium* was also reported in laboratory-reared cynomolgus monkeys (0.5%) in Guangxi, China [22]. In addition, the prevalence of *Cryptosporidium* in *M. fascicularis* in the present study was higher than that reported for *M. fascicularis* in Thailand (1.0%), mountain gorillas in Rwanda (4.0%) and Uganda (4.0%), in orangutans from Indonesia (2.7%), in olive baboons from Kenya (2.6%), and in western lowland gorillas from the Central African Republic (0.5%) [16, 17, 23–25, 27]. However, although the reported positivity rates were lower in wild primates conducted in other countries compared to the present study, NHPs from Tanzania exhibited much higher rates of infection than those observed here [26]. The differences in prevalence may be related to factors such as differences in regional environments, the sensitivity and specificity of detection methods, animal the health statuses of the animals at the time of sampling, and the overall sample size. We also observed that the infection rate of *Cryptosporidium* in *M. fascicularis* (5.7%) was higher than that in *M. mulatta* (0%). In consideration of this observation, as well as those mentioned above, the species of NHPs may reflect differences in susceptibility, which could explain the species-to-species variation in prevalence. In fact, 64.8% of the *M. fascicularis* in the present study were less than one year-old, whereas all of the *M. mulatta* were over 10 years of age. For *M. fascicularis*, infection rates of young animals were higher than that of adult animals. These data suggest that age may be a risk factor in NHPs. In support of this conclusion, another study has reported that infant baboons were infected at a higher rate (15.4%) than that of adults (1.3%) [15].

In the present study, only *C. hominis* was detected in *M. fascicularis*. Although there are other reports of *Cryptosporidium* spp. in non-human primates, few have provided detailed molecular information about these parasites in the great apes (Table 2) [15, 17–29]. Previous studies have indicated that at least eight species of *Cryptosporidium* have been reported in NHPs, including *C. hominis*, *C. parvum*, *C. felis*, *C. muris*, *C. bovis*, *C. ubiquitum*, *C. meleagridis* and *C. andersoni*. Among these, *C. hominis* was the most common *Cryptosporidium* species detected in NHPs (80/120) (Table 2) [15, 17–29]. The potential for zoonotic transmission is evident for the species and subtypes detected. *C. hominis* was the most common species in human cases of cryptosporidiosis in China, accounting for 48.3% (127/263) of all cryptosporidiosis cases [32]. Although *C. hominis* is widely considered a human-specific pathogen, and humans are the only natural host, it is a rather common *Cryptosporidium* species detected in non-human primates, horses and donkeys [10]. Furthermore, *C. hominis* has recently been identified in cattle, yaks, sheep, goats, kangaroos, rodents, hedgehogs, dogs and dugong [10]. Experimental infections with *C. hominis* have been successful in Mongolian gerbils, piglets and mouse [33–35]. The true and natural host range of *C. hominis* needs to be confirmed by subsequent molecular data from epidemiological studies of *Cryptosporidium*.

**Table 2:** Prevalence and distribution of *Cryptosporidium* species and subtypes in natural infection of non human primates worldwide

Country	Species of NHPs	No. positive/no. examined (%)	Species (*n*)	Subtype (*n*)^a^	Ref
Central African Republic	*Gorilla gorilla gorilla* (western lowland gorillas)	1/201 (0.5)	*C. bovis* (1)	–	[17]
China	*Saimiri sciureus* (squirrel monkey)	1	*C. hominis* (1)	IkA7G4 (1)^b^	[18]
China	*Macaca mulatta* (rhesus macaque)	6/86 (7.0)	*C. andersoni* (5); *C. parvum* (1)	IIdA15G2R1 (1)	[19]
China	*Macaca mulatta* (rhesus macaque)	9/1316 (0.7)	*C. hominis* (9)	IbA12G3 (7); PN (2)	[20]
China	*Macaca fascicularis* (long-tailed macaque)	8/778 (1.0)	*C. muris* (4); *C. hominis* (4)	IiA17 (1); PN (3)	[20]
China	*Nycticebus bengalensis* (Bengal slow loris)	1/10 (10.0)	*C. muris* (1)	–	[20]
China	*Presbytis francoisi* (Francoisʼ leaf monkey)	1/15 (6.7)	*C. hominis* (1)	pn	[20]
China	*Macaca mulatta* (rhesus macaque)	45/411 (10.9)	*C. hominis* (39); *C. parvum* (5); *C. felis* (1)	IaA13R7 (2); IaA13R8 (6); IaA14R7 (1); IdA20 (10); IeA11G3T3 (8); IfA16G2 (1); IIcA5G3a (5)	[21]
China	*Macaca fascicularis* (long-tailed macaque)	1/205 (0.5)	*C. hominis* (1)	IdA14 (1)	[22]
Indonesia	*Pongo abelii*; *Pongo pygmaeus* (orangutans)	8/298 (2.7)	*C. parvum* (2); *C. muris* (6)	–	[23]
Kenya	*Papio anubis* (olive baboon)	6/235 (2.6)	*C. hominis* (6)	IfA12G2 (2); IbA9G3 (2); IiA14 (1)	[15]
Rwanda	*Gorilla beringei beringei* (mountain gorilla)	4/100 (4.0)	*C. muris* (2); *C. meleagridis* (2)	–	[24]
Thailand	*Macaca fascicularis* (long-tailed macaque)	2/200 (1.0)	*C. hominis* (2)	–	[25]
Tanzania	*Papio anubis* (olive baboon)	5/47 (10.6)	*C. hominis* (5)	IfA12G2 (3/5)	[26]
Tanzania	*Pan troglodytes* (chimpanzee)	12/58 (20.7)	*C. hominis* (7); *C. suis* (6)	IfA12G2 (2/7)	[26]
Tanzania	*Pan troglodytes* (chimpanzee)	4/26 (15.4)	*C. hominis* (4)	IfA12G2 (3/4)	[26]
Uganda	*Gorilla beringei beringei* (mountain gorilla)	4/100 (4.0)	*C. parvum* (4)	–	[27]
USA	*Propithecus coquereli* (Coquerel’s sifaka)	1	*C. suis* (1)	–	[28]
USA	*Macaca mulatta* (rhesus macaque)	1	*C. hominis* (1)	IiA17	[29]

^a^ The numbers of subtypes are not consistent with the positives of *C. hominis* in some countries because not all isolates were genotyped successfully

^b^ Subtype IkA7G4 was misnamed

*Abbreviations*: Ref, reference; pn, PCR-negative; −, not subtyping or no subtypes

To date, more than 10 subtype families have been recognized in *C. hominis* based on sequence analysis of the *gp60* gene; six of these subtype families (Ia, Ib, Id, Ie, If and Ii) have been identified in NHPs (Table 2) [15, 17–29]. Subtype families Ia, Ib, Id and Ie account for the majority of worldwide cases in humans [10]. In the present study, three subtype families (Ia, Io, and Ii) were identified, and were composed of IaA20R3a, IoA17a, IoA17b and IiA17. Among them, subtype IiA17 was the dominant subtype (66.7%) which has been reported in a rhesus monkey from the USA, in a crab-eating macaque from China, and two humans from Sweden who had visited a monkey farm in Thailand [20, 29, 36]. The subtypes IaA20R3a, IoA17a and IoA17b identified here have not been reported to the best of our knowledge, thus representing new subtypes. The subtype family Ia is common in humans from China, and IaA14R4 was the cause of a cryptosporidiosis outbreak in a pediatric ward in Shanghai [37]. In fact, *C. hominis* Ia subtype families have also been detected at high frequencies in rhesus monkeys from Guizhou, China. Thus, NHPs can play a potential role in zoonotic transmission of *C. hominis* in China [21]. Also of concern, the *gp60* subtype family Io, and the related Id, have been identified in two horses in China [38]. The new subtypes IoA17a and IoA17b found in *M. fascicularis* here, is possibly a reflection of endemic transmission of *C. hominis* in these animals. The true subtype constitutions of *C. hominis* in *M. fascicularis* need to be confirmed by more systematic epidemiological studies of *Cryptosporidium* from these animals in the future.

## Conclusions

In this study, we determined the prevalence rate of *C. hominis* among *M. fascicularis* in a breeding base of experimental primates in Hainan Island of China. The novel subtypes of *C. hominis* detected in *M. fascicularis* might present the endemic genetic characterization of population structure of *Cryptosporidium*, although the genetic diversity among *C. hominis* subtypes is not well understood. To better evaluate the transmission of *Cryptosporidium* from *M. fascicularis* to humans, more studies investigating the biology, population genetics and transmission dynamics of *Cryptosporidium* in *M. fascicularis* throughout different geographical regions are needed.

## Abbreviations

NHPs: non-human primates
PCR: polymerase chain reaction
*gp60*: 60-kilodalton glycoprotein
*SSU* rRNA: small subunit of nuclear ribosomal RNA
HIV: human immunodeficiency virus
AIDS: Acquired Immunodeficiency Syndrome
NIH: National Institutes of Health
FAO: Food and Agriculture Organization
WHO: World Health Organization

## References

[1] Checkley W, White AC, Jaganath D, Arrowood MJ, Chalmers RM, Chen XM (2015). A review of the global burden, novel diagnostics, therapeutics, and vaccine targets for *Cryptosporidium*. Lancet Infect Dis..

[2] Colford JM, Tager IB, Hirozawa AM, Lemp GF, Aragon T, Petersen C (1996). Cryptosporidiosis among patients infected with human immunodeficiency virus. Factors related to symptomatic infection and survival. Am J Epidemiol..

[3] GBD Diarrhoeal Diseases Collaborators (2017). Estimates of global, regional, and national morbidity, mortality, and aetiologies of diarrhoeal diseases: a systematic analysis for the Global Burden of Disease Study 2015. Lancet Infect Dis..

[4] Ryan U, Hijjawi N (2015). New developments in *Cryptosporidium* research. Int J Parasitol..

[5] Chalmers RM, Giles M (2010). Zoonotic cryptosporidiosis in the UK—challenges for control. J Appl Microbiol..

[6] Efstratiou A, Ongerth JE, Karanis P (2017). Waterborne transmission of protozoan parasites: review of worldwide outbreaks - an update 2011–2016. Water Res..

[7] Ryan U, Hijjawi N, Xiao L (2018). Foodborne cryptosporidiosis. Int J Parasitol..

[8] NIAID Emerging Infectious Diseases/Pathogens. (https://www.niaid.nih.gov/research/emerging-infectious-diseases-pathogens). Accessed 10 Dec 2018.

[9] Report of a Joint FAO/WHO Expert Meeting, 3–7 September 2012, FAO Headquarters, Rome, Italy. (http://www.fao.org/food/food-safety-quality/a-zindex/foodborne-parasites/en/). Accessed 10 Dec 2018.

[10] Feng Y, Ryan UM, Xiao L (2018). Genetic diversity and population structure of *Cryptosporidium*. Trends Parasitol..

[11] Khan A, Shaik JS, Grigg ME (2018). Genomics and molecular epidemiology of *Cryptosporidium* species. Acta Trop..

[12] Xiao L, Feng Y (2017). Molecular epidemiologic tools for waterborne pathogen*s Cryptosporidium* spp. and *Giardia duodenalis*. Food Waterborne Parasitol..

[13] Chalmers RM, Smith RP, Hadfield SJ, Elwin K, Giles M (2011). Zoonotic linkage and variation in *Cryptosporidium parvum* from patients in the United Kingdom. Parasitol Res..

[14] Cacciò SM, Sannella AR, Mariano V, Valentini S, Berti F, Tosini F (2013). A rare *Cryptosporidium parvum* genotype associated with infection of lambs and zoonotic transmission in Italy. Vet Parasitol..

[15] Li W, Kiulia NM, Mwenda JM, Nyachieo A, Taylor MB, Zhang X (2011). *Cyclospora papionis*, *Cryptosporidium hominis*, and human-pathogenic *Enterocytozoon bieneusi* in captive baboons in Kenya. J Clin Microbiol..

[16] Kváč M, McEvoy J, Stenger B, Clark M, Cacciò SM, Widmer G (2014). Cryptosporidiosis in other vertebrates. *Cryptosporidium*: parasite and disease.

[17] Sak B, Petrzelkova KJ, Kvetonova D, Mynarova A, Shutt KA, Pomajbikova K (2013). Long-term monitoring of microsporidia, *Cryptosporidium* and *Giardia* infections in western Lowland Gorillas (*Gorilla gorilla gorilla*) at different stages of habituation in Dzanga Sangha Protected Areas, Central African Republic. PLoS ONE.

[18] Liu X, Xie N, Li W, Zhou Z, Zhong Z, Shen L (2015). Emergence of *Cryptosporidium hominis* monkey genotype II and novel subtype family Ik in the squirrel monkey (*Saimiri sciureus*) in China. PLoS ONE.

[19] Du SZ, Zhao GH, Shao JF, Fang YQ, Tian GR, Zhang LX (2015). *Cryptosporidium* spp., *Giardia intestinalis*, and *Enterocytozoon bieneusi* in captive non-human primates in Qinling Mountains. Korean J Parasitol..

[20] Karim MR, Zhang S, Jian F, Li J, Zhou C, Zhang L (2014). Multilocus typing of *Cryptosporidium* spp. and *Giardia duodenalis* from non-human primates in China. Int J Parasitol..

[21] Ye J, Xiao L, Ma J, Guo M, Liu L, Feng Y (2012). Anthroponotic enteric parasites in monkeys in public park, China. Emerg Infect Dis..

[22] Ye J, Xiao L, Li J, Huang W, Amer SE, Guo Y (2014). Occurrence of human-pathogenic *Enterocytozoon bieneusi*, *Giardia duodenalis* and *Cryptosporidium* genotypes in laboratory macaques in Guangxi, China. Parasitol Int..

[23] Mynářová A, Foitová I, Kváč M, Květoňová D, Rost M, Morrogh-Bernard H, Nurcahyo W (2016). Prevalence of *Cryptosporidium* spp., *Enterocytozoon bieneusi*, *Encephalitozoon* spp. and *Giardia intestinalis* in wild, semi-wild and captive orangutans (*Pongo abelii* and *Pongo pygmaeus*) on Sumatra and Borneo, Indonesia. PLoS ONE..

[24] Sak B, Petrželková KJ, Květoňová D, Mynářová A, Pomajbíková K, Modrý D (2014). Diversity of microsporidia, *Cryptosporidium* and *Giardia* in mountain gorillas (*Gorilla beringei beringei*) in Volcanoes National Park, Rwanda. PLoS ONE..

[25] Sricharern W, Inpankaew T, Keawmongkol S, Supanam J, Stich RW, Jittapalapong S (2016). Molecular detection and prevalence of *Giardia duodenalis* and *Cryptosporidium* spp. among long-tailed macaques (*Macaca fascicularis*) in Thailand. Infect Genet Evol..

[26] Parsons MB, Travis D, Lonsdorf EV, Lipende I, Roellig DM, Collins A (2015). Epidemiology and molecular characterization of *Cryptosporidium* spp. in humans, wild primates, and domesticated animals in the Greater Gombe Ecosystem, Tanzania. PLoS Negl Trop Dis..

[27] Graczyk TK, DaSilva AJ, Cranfield MR, Nizeyi JB, Kalema GR, Pieniazek NJ (2001). *Cryptosporidium parvum* genotype 2 infections in free-ranging mountain gorillas (*Gorilla gorilla beringei*) of the Bwindi Impenetrable National Park, Uganda. Parasitol Res..

[28] da Silva AJ, Cacciò S, Williams C, Won KY, Nace EK, Whittier C (2003). Molecular and morphologic characterization of a *Cryptosporidium* genotype identified in lemurs. Vet Parasitol..

[29] Feng Y, Lal AA, Li N, Xiao L (2011). Subtypes of *Cryptosporidium* spp. in mice and other small mammals. Exp Parasitol..

[30] Xiao L, Escalante L, Yang C, Sulaiman I, Escalante AA, Montali RJ (1999). Phylogenetic analysis of *Cryptosporidium* parasites based on the small-subunit rRNA gene locus. Appl Environ Microbiol..

[31] Alves M, Xiao L, Sulaiman I, Lal AA, Matos O, Antunes F (2003). Subgenotype analysis of *Cryptosporidium* isolates from humans, cattle, and zoo ruminants in Portugal. J Clin Microbiol..

[32] Feng Y, Xiao L (2017). Molecular epidemiology of *Cryptosporidiosis* in China. Front Microbiol..

[33] Baishanbo A, Gargala G, Delaunay A, François A, Ballet JJ, Favennec L (2005). Infectivity of *Cryptosporidium hominis* and *Cryptosporidium parvum* genotype 2 isolates in immunosuppressed Mongolian gerbils. Infect Immun..

[34] Roche JK, Rojo AL, Costa LB, Smeltz R, Manque P, Woehlbier U (2013). Intranasal vaccination in mice with an attenuated *Salmonella enterica* Serovar 908htr A expressing Cp15 of *Cryptosporidium*: impact of malnutrition with preservation of cytokine secretion. Vaccine..

[35] Lee S, Ginese M, Beamer G, Danz HR, Girouard DJ, Chapman-Bonofiglio SP (2018). Therapeutic efficacy of bumped kinase inhibitor 1369 in a pig model of acute diarrhea caused by *Cryptosporidium hominis*. Antimicrob Agents Chemother..

[36] Lebbad M, Winiecka-Krusnell J, Insulander M, Beser J (2018). Molecular characterization and epidemiological investigation of *Cryptosporidium hominis* IkA18G1 and *C. hominis* monkey genotype IiA17, two unusual subtypes diagnosed in Swedish patients. Exp Parasitol..

[37] Feng Y, Wang L, Duan L, Gomez-Puerta LA, Zhang L, Zhao X (2012). Extended outbreak of cryptosporidiosis in a pediatric hospital, China. Emerg Infect Dis..

[38] Deng L, Li W, Zhong Z, Gong C, Cao X, Song Y (2017). Occurrence and genetic characteristics of *Cryptosporidium hominis* and *Cryptosporidium andersoni* in horses from southwestern China. J Eukaryot Microbiol..

